# Reply to Augello, P.A.; Wu, J. Comment on “Rogers et al. The Combined Effects of Cannabis, Methamphetamine, and HIV on Neurocognition. *Viruses* 2023, *15*, 674”

**DOI:** 10.3390/v15091878

**Published:** 2023-09-05

**Authors:** Jeffrey M. Rogers, Jennifer E. Iudicello, Maria Cecilia G. Marcondes, Erin E. Morgan, Mariana Cherner, Ronald J. Ellis, Scott L. Letendre, Robert K. Heaton, Igor Grant

**Affiliations:** 1 San Diego State University/University of California San Diego Joint Doctoral Program in Clinical Psychology, San Diego, CA 92120, USA; 2 Department of Psychiatry, University of California San Diego, San Diego, CA 92093, USA; 3 San Diego Biomedical Research Institute, San Diego, CA 92121, USA; 4 Department of Neurosciences, University of California San Diego, San Diego, CA 92093, USA; 5 Department of Medicine, University of California San Diego, San Diego, CA 92093, USA

We thank Augello and Wu [1] for their interest in our manuscript and for bringing to our attention these inconsistencies between the modeled slopes and the group means presented in the figures. We regret that this did not come to our attention sooner, and we have gone about trying to determine the source of these discrepancies. Your point that there should be concordance between the group mean differences (whether as modeled T-scores or probabilities of impairment) derived from the figures and the model slope estimates presented in the tables is well taken. These should be parallel to one another. After re-examining the analysis syntax and implementing another contrast coding strategy with the same comparisons (backward deviance coding), but which are specified in different directions (see below), estimates matched the fitted values displayed in the profile plots. The corrected results are largely consistent with the main conclusions originally reported in the manuscript; namely, that M+C+ displayed better neurocognitive performance and lower rates of impairment than M+C−. The corrected results are different from those reported in the manuscript in terms of the effect size reported. Additionally, the corrected results now support the notion that M+C+ did not display a worse performance than a comparison group without these substance use disorder histories (M−C−). People with only cannabis use disorder histories (M−C+) still displayed better overall performance than M−C−, but they did so in different subdomains than originally reported. All corrections discussed have been addressed in a Correction submitted by the authors.

The directions specified in our first contrast codes appear to have caused parameter estimation issues, though both sets of contrasts below produce the same model-fitted values, and other model estimates appear to remain unchanged.


**Original**
CorrectedContrast #1—M+C+ to M+C−Contrast #1—M+C− to M+C+Contrast #2—M+C+ to M−C−Contrast #2—M+C+ to M−C−Contrast #3—M−C+ to M−C−Contrast #3—M−C− to M−C+

Models were re-specified using a contrast code set that was constructed using backward difference coding [2].



**Level 1 vs. Level 2**

**Level 2 vs. Level 3**

**Level 3 vs. Level 4**

M/C Groups
M+C− to M+C+M+C+ to M−C−M−C− to M−C+M+C−−(k − 1)/k−(k − 2)/k−(k − 3)/kM+C+
1/k
−(k − 2)/k−(k − 3)/kM−C−
1/k

2/k
−(k − 3)/kM−C+
1/k

2/k

3/k


Models examining associations between cannabis and methamphetamine characteristics’ associations with neurocognitive performance and impairment were not impacted by the contrast coding error, as only the two groups with a lifetime for cannabis or methamphetamine were compared in each set.

Models examining interactions between the substance use group and HIV status/characteristics were not impacted by the contrast codes operationalization error, as contrasts were comparison coded vs. M−C− in these models to aid in the interpretation of model interactions. However, the main effects model without interactions presented in Supplementary Table S3 of the original manuscript needed to be re-estimated, and these changes are reflected in the Correction submitted by the authors. Changes to model estimates did not impact inferences discussed in the manuscript and, like in other models, the same fitted values were produced as previously described.
